# Residual characteristics and safety assessment of the insecticides spiromesifen and chromafenozide in lettuce and perilla

**DOI:** 10.1038/s41598-022-08532-2

**Published:** 2022-03-18

**Authors:** Syed Wasim Sardar, Geon Doo Byeon, Jeong Yoon Choi, Hun Ju Ham, Abd Elaziz Sulieman Ahmed Ishag, Jang Hyun Hur

**Affiliations:** 1 Department of Biological Environment, Kangwon National University, Chuncheon, 24341 Republic of Korea; 2 Department of Crop Protection, University of Khartoum, 13314 Khartoum North, Shambat, Sudan

**Keywords:** Biochemistry, Ecology, Ecology, Environmental sciences

## Abstract

This study was performed to investigate the residual characteristics, safety assessment, and pre-harvest interval (PHI) of spiromesifen and chromafenozide in lettuce (*Latuca sativa* L.) and perilla (*Perilla frutescens* (L.) Britton) leaves. Samples were harvested periodically, extracted using QuEChERS method, and analyzed by LC-MS/MS. Average recoveries of spiromesifen and its metabolite BSN2060-enol and chromafenozide were ranged from 80.6 to 107.9%, with relative standard deviation < 10%. Spiromesifen and cromafenozide initial residues in lettuce were dissipated to 81.45 and 95.52% after 7 days, with half-lives of 2.89 and 1.69 days respectively. Values in perilla leaves were 76.68 and 61.27% after the same period, with half-lives of 4.25 and 6.30 days, respectively. Risk assessment results showed that %ADI (acceptable daily intake) of spiromesifen and chromafenozide was 6.83 and 0.56, in lettuce and 4.60 and 0.25% in perilla leaves, respectively. Theoretical maximum daily intakes of spiromesifen and chromafenozide were 67.49 and 3.43%, respectively, indicating that residues of both compounds pose no considerable health risks to consumers. This study provides data for setting maximum residue limits and PHIs for the safe use of spiromesifen and chromafenozide in lettuce and perilla.

## Introduction

Crops cultivated in a land area of smaller than 1000 hectares are classified as minor crops^1^. Although minor crops are grown on a small scale, they generally yield substantial economic profits to several countries^2^. However, it is difficult to control pests during the cultivation of minor crops due to the lack of registered pesticides for each minor crop^1^. In general, minor crops do not provide an adequate economic return for pesticide manufacturers to justify the cost of registration^3^. As a result, farmers who grow minor crops, use the existing or unregistered pesticides for pest control^4^. Further, unregistered pesticide residues in crops are of great concern due to their potential health risk^5^.

The minor crops lettuce (*Latuca sativa* L.) and perilla (*Perilla frutescens* (L.) Britton) are one of the most important components of the human diet in several countries such as Korea, China, and Japan, where it is consumed in its raw form as a salad, food wrap or home-cooked^6,7^. As the consumption of lettuce and perilla leaves has increased in Republic of Korea, farmers have started cultivating the crop in greenhouses. However, due to the lack of registered pesticides for the target crops, farmers are having difficulties in controlling several types of insect pests such as spider mites and lepidopteran.

In this study spiromesifen (3-mesityl-2-oxo-1-oxaspiro[4.4]non-3-en-4-yl 3,3-dimethylbutyrate) and chromafenozide (2-*tert*-butyl-5-methyl-2-(3,5-xyloyl) chromane-6-carbohydrazide) were selected as test insecticides for lettuce and perilla crops. Spiromesifen is a novel non-systemic insecticide from the new chemical class of tetronic acid derivatives that act effectively against whiteflies (*Bemisia* spp. and *Trialeurodes* spp.) and tetranychid spider mite species (*Tetranychus* spp.)^8^. It controls the growth of larva through the inhibition of the lipid metabolism enzyme (acetyl CoA-carboxylase) and causes a significant decrease in the lipids^9^. In addition, it does not show cross-resistance against mites and whiteflies, and thus has excellent insecticidal efficacy against insects^10^. Chromafenozide is a novel dibenzoylhydrazine insecticide that controls lepidopteran pests on various crops such as rice, vegetables, and fruit trees. The mode of action of chromafenozide is the inhibition of insect growth regulator, mainly disturbing the normal growth and development of an insect, causing incomplete and lethal moult^10,11^. To the best of our knowledge, there are no reports available on the dissipation patterns and residual characteristics of spiromesifen and chromafenozide in lettuce and perilla leaves. Moreover, in the Republic of Korea, there was no maximum residue limit (MRL) of spiromesifen and chromafenozide for lettuce and perilla leaves, when this study was performed. Therefore, this study aimed (1) to investigate the dissipation pattern, residual characteristics of spiromesifen and chromafenozide in lettuce and perilla leaves under greenhouse farm, (2) to provide the basic data for the establishment of MRLs and pre-harvest intervals (PHI) for the safe use of the two insecticides during the cultivation of the target crops, (3) to evaluate estimated daily intake (EDI) and theoretical maximum daily intake (TMDI) for Korean consumers by using residue data, MRLs, food factors, and correction factors, and compared with the acceptable daily intake (ADI) to estimate the risks of the pesticide residues to human health. The result of this study provides a scientific basis for assessing the residual characteristics and safety of pesticide residues in leafy vegetables.

## Result and discussion

### Method validation

The validation of the analytical method was carried out by evaluating different parameters including linearity, accuracy, precision, and method limits of quantification (MLOQ). The linearity of the seven-point matrix-matched calibration curve over the range of 0.01 to 1.0 mg/kg was excellent with a regression coefficient (R^2^) of 0.99 for all compounds (Fig S1 and S2). The recoveries of spiromesifen, BSN2060-enol, and chromafenozide at 0.1 and 0.5 mg/kg fortification levels ranged between 88.2 to 101.9%, 97.3 to 103.2%, and 95.6 to 100.8%, respectively, in lettuce (Table S1). The respective values in perilla leaves were 78.0 to 99.9%, 77.8 to 87.5%, and 99.4 to 113.5% (Table S1). The repeatability expressed as the RSD of the analyzed samples (n = 3) was below 10% for all compounds. The MLOQ of all analytes was 0.01 mg/kg. There were no interfering peaks found around the retention times of the target compounds in spiked and control samples, showing that the method was specifically accurate. The MLOQs and average recovery data of the test compounds are shown in Table 1.

**Table 1:** Average recoveries and MLOQs of insecticides in lettuce and perilla leaves.

Insecticides	Crops	Fortification level (mg/kg)	Recoveries ± RSD (%)	MLOQ (mg/kg)
Spiromesifen	Lettuce	0.1	99.2 ± 3.1	0.01
		0.5	94.5 ± 7.3	
	Perilla leaves	0.1	94.6 ± 5.0	
		0.5	81.3 ± 5.7	
BSN2060-enol	Lettuce	0.1	98.7 ± 1.3	
		0.5	100.1 ± 2.9	
	Perilla leaves	0.1	80.6 ± 3.4	
		0.5	82.5 ± 5.2	
Chromafenozide	Lettuce	0.1	99.9 ± 0.4	
		0.5	98.3 ± 2.7	
	Perilla leaves	0.1	102.0 ± 2.7	
		0.5	107.9 ± 4.5	

*MLOQ* method limit of quantitation, *RSD* relative standard deviation.

### Initial deposition and dissipation characteristics of insecticides

The initial residue is a theoretical concentration of insecticides that could be deposited on crops. The calculation was carried out based on the 0-day residues (residue detected after two hours of insecticides application), considering the active ingredient concentration percentage in the pesticide formulation, dilution factors, and type of the formulations used^12^. The results in Table 2 indicated that the initial residues of spiromesifen and chromafenozide in lettuce were 136.90 and 107.20 mg/kg, respectively. While the initial residues of spiromesifen and chromafenozide in perilla leaves were 189.75 and 136.90 mg/kg, respectively. In both crops, the initial residues of spiromesifen were slightly higher than that of chromafenozide. These findings agree with previous studies in the context that the initial residues of pesticides in crops depend on several factors such as the active ingredient (AI), formulation, dilution factor, and type of crop^13^. The AI used in this study was spiromesifen SC 20% and chromafenozide EC 5%, it may be assumed that the higher initial residues of spiromesifen are due to the high concentration of AI present in the formulation products^4^. Pesticide formulation has major effects on its penetration through the leaf surface and dissipation. Generally, SC formulations retained better than wettable powder (WP) form^4^. The insecticide formulations used in the current study were spiromesifen SC and chromafenozide EC, which are considered to have good adhesion and easy penetration into the crops contributing to high initial residues^14^. Moreover, spiromesifen and chromafenozide were applied on lettuce and perilla leaves under similar environmental conditions, however, the initial residues in perilla leaves were higher than that of lettuce, which might be attributed to the hairy leaf of the perilla plant^15^.

**Table 2:** Initial residues of spiromesifen^a^ and chromafenozide in lettuce and perilla leaves.

Crops	Insecticides	Residual amount at 0 day (mg/kg)	Initial residual amount (mg/kg)
Lettuce	Spiromesifen	27.38	136.90
	Chromafenozide	10.72	107.20
Perilla leaves	Spiromesifen	37.95	189.75
	Chromafenozide	12.55	136.90

^a^Total residues of spiromesifen (mg/kg) = spiromesifen + (BSN2060 residue × 1.36).

$${1}.{36}\,({\text{conversion}}\,{\text{factor}}) = \frac{{370.49\,\left( {{\text{spiromesifen}}\,{\text{MW}}} \right)}}{{272.34\,\left( {{\text{BSN}}2060\,{\text{MW}}} \right)}}$$.

**Figure 1:**
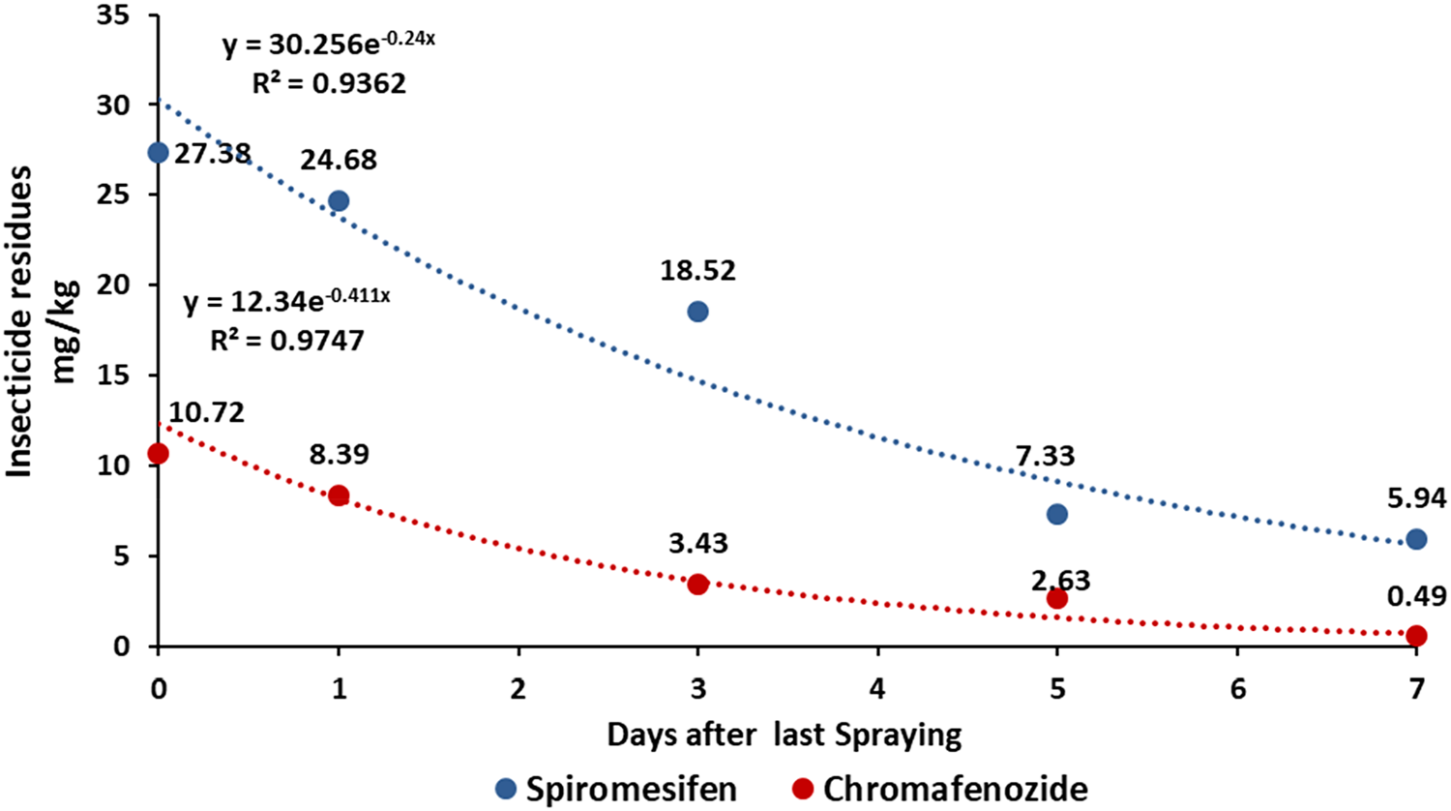
Dissipation patterns and half-lives of insecticides in lettuce.

**Figure 2:**
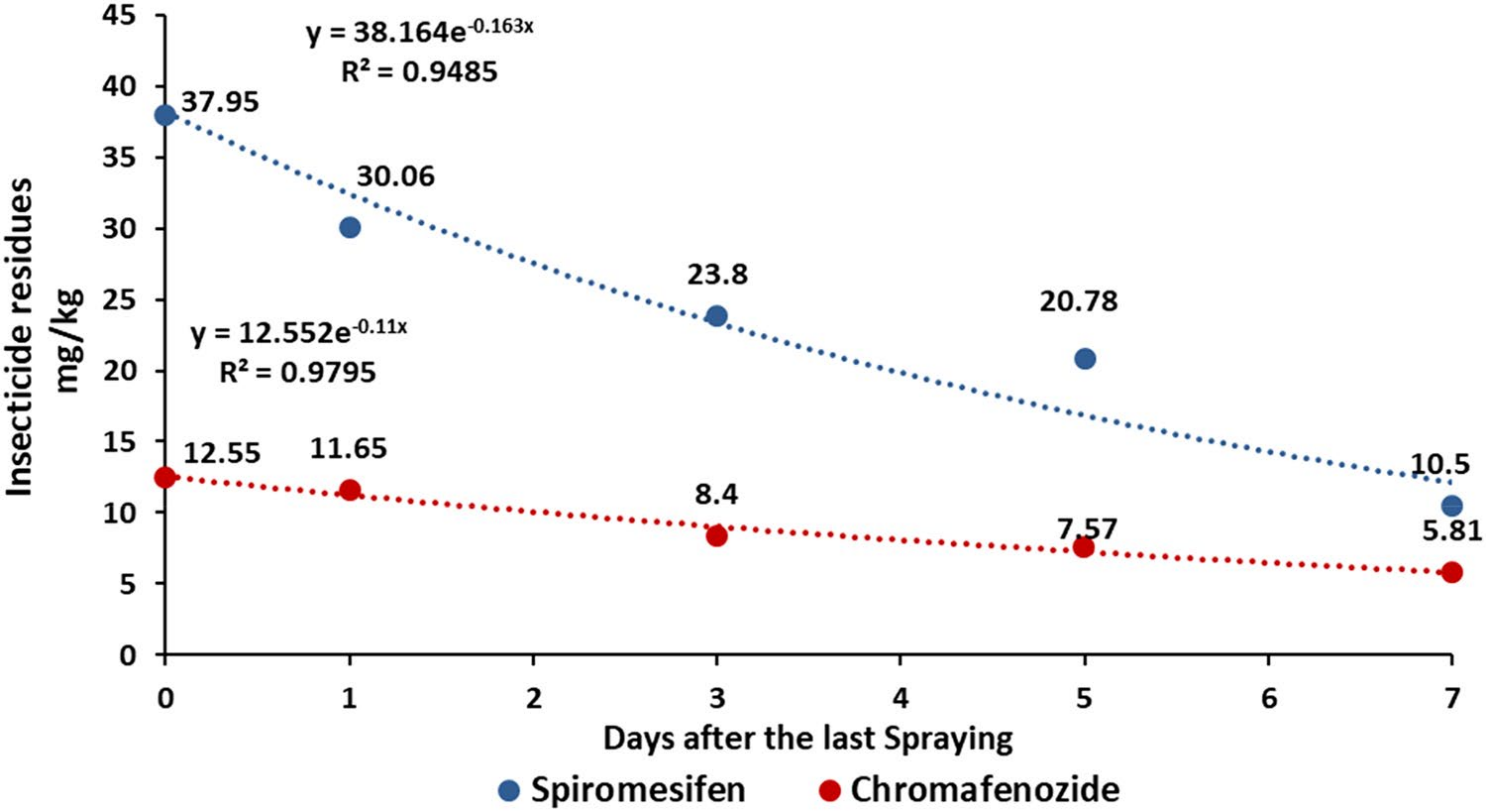
Dissipation patterns and half-lives of insecticides in perilla leaves.

The dissipation rate and half-lives of pesticides provide an important index to assess the behavior in plants. The dissipation of spiromesifen and chromafenozide in lettuce and perilla leaves were found following the first-order kinetics model with a correlation coefficient between 0.93 and 0.97 (Figs. 1, 2). As the duration from last insecticide application to sampling time increased, spiromesifen and chromafenozide residue levels decreased. The initial residues of spiromesifen and cromafenozide in lettuce were dissipated to an extent of 81.45 and 95.52% after 7 days, with the half-lives of 2.89 and 1.69 days, respectively. Similarly, the initial residues of spiromesifen and cromafenozide in perilla leaves were dissipated to 76.68 and 61.27% after 7 days, with the half-lives of 4.25 and 6.30 days, correspondingly. The half-life period of spiromesifen and cromafenozide in lettuce and perilla leaves was less than a week. Sharma et al.^16^ and Chauhan et al.^17^ have observed a similar pattern of spiromesifen and cromafenozide half-life. They reported that spiromesifen have a half-life of 5.64 days in cucumber, 3 days in eggplant, 5 to 6 days in apple, and 5 days in tea^16,17^. In other studies, chromafenozide has been reported to have a half-life of 3.53 to 4.07 days in strawberries and 3.5 days in tomatoes^10,18^. The dissipation of pesticides in plants is affected by various factors including, physicochemical properties, initial concentration, characteristics of the crop species, frequency of application, and environmental factors^19,20^. Besides the degradation of the pesticide itself due to environmental factors, the growth-dilution effect of crops plays a substantial role in dissipation^14^. Several studies have reported that the dilution effect caused by crop growth is a major factor that contributes to the decline of pesticides^19^. In another study, Jeon et al.^21^ discovered that the decreasing residual level of bifenthrin in perilla leaves over time was due to the growth of the plant. Furthermore, Kim et al.^22^ reported that the half-lives of chlorpyrifos and procymidone in lettuce were 1.2 to 1.5 days and 1.3 to 2.6 days, respectively and the major cause of the decrease over time was the growth dilution factor. In this study, although spiromesifen and chromafenozide were similarly applied to lettuce and perilla leaves, the rate of degradation of both insecticides in lettuce was faster than that in perilla leaves. Such differences could be correlated to the growth characteristics of the two crops^15^. This result suggests that the dissipation and half-life of pesticide residues can vary based on crop variety.

### Residual characteristics of insecticides

To obtain a proper protection regime, spiromesifen and chromafenozide were sprayed twice on lettuce and perilla leaves. The insecticides residue amount at various growth stages (0, 1, 3, 5, and 7 days after spraying) of lettuce and perilla were measured and the results are summarized in Table 3. The residues of spiromesifen were calculated as the sum of spiromesifen and its metabolite BSN2060-enol following RDA guidelines^23^. The residues of spiromesifen and chromafenozide in lettuce after 7 days were decreased from 27.07 to 5.50 mg/kg and 9.30 to 0.48 mg/kg, respectively. Similarly, perilla leaves after the same periods were decreased from 34.52 to 9.67 mg/kg (spiromesifen) and 12.40 to 5.26 mg/kg (chromafenozide). Several factors affect the residual characteristics of pesticides such as crop morphology, growth rate, and physicochemical properties of pesticides^14,24^. In this study, although the tendency of spiromesifen and chromafenozide to decrease over time was similar, the residual amounts of both compounds in perilla leaves were relatively higher than that of lettuce. This higher residual amount may be due to the morphological features of the crops, as perilla has hairy leaves while lettuce leaves had deep curves^25^. Thus, compared to curved leaves, the foliar sprays deposit a high amount of insecticide on hairy leaves^26^. The result of the present study is consistent with the previous study in which the amount of pesticide in hairy peaches was 1.9 times higher than that of without hair^27^. Moreover, a faster growth rate of crop increases the contribution of growth dilution to pesticide dissipation^14^. Hwang et al.^28^ described that the wider leaf and rapid growth of lettuce consider as another factor that influences the residue of pesticides^28^. Another factor affecting the residual characteristics of pesticide are the physicochemical properties^29,30^. The log P value of pesticide is used as an index to predict the lipophilicity, and it has been reported that pesticides with a log P value greater than 1.0 have a good penetration property^31,32^. In the case of spiromesifen and chromafenozide the log P values were 4.55 and 2.7 respectively, which are considered to be easily penetrating the crop^33^. Therefore, it is assumed that the higher residues of spiromesifen detected in lettuce and perilla leaves are due to its high concentration of AI in the formulation products as well as 100-time higher log P value. Our results are consistent with the results of previous studies, which have reported that the initial residues, residual patterns, and biological half-life of pesticides are affected by crop morphology and physicochemical characteristics of pesticides^14,24,34^.

**Table 3:** Residual amounts of spiromesifen^a^ and chromafenozide in lettuce and perilla leaves.

Crops	Insecticides	Days after the last spraying	Average residual amount (mg/kg)
Lettuce	Spiromesifen	0	27.07
		1	23.13
		3	16.55
		5	6.79
		7	5.50
	Chromafenozide	0	9.30
		1	7.25
		3	2.78
		5	2.08
		7	0.48
Perilla leaves	Spiromesifen	0	34.52
		1	26.08
		3	22.72
		5	18.64
		7	9.67
	Chromafenozide	0	12.40
		1	10.22
		3	8.21
		5	6.58
		7	5.26

^a^Total residues of spiromesifen (mg/kg) = spiromesifen + (BSN2060 residue × 1.36).

$${1}.{36}\,({\text{conversion}}\,{\text{factor}}) = \frac{{370.49\,\left( {{\text{spiromesifen}}\,{\text{MW}}} \right)}}{{272.34\,\left( {{\text{BSN}}2060\,{\text{MW}}} \right)}}$$

### Safety assessment

The %ADI of spiromesifen and chromafenozide in lettuce and perilla leaves consumed by Koreans are shown in Table 4. Based on the data provided by the Korean Health Industry Development Institute (KHIDI)^35^, on average, 0.00615 kg/day of lettuce and 0.00276 kg/day of perilla leaves are consumed by the average Korean adult (60 kg weight). The ADI of spiromesifen set by the Korean authorities is 0.03 mg/kg/body weight/day, and chromafenozide is 0.27 mg/kg/bodyweight/day^36^. The %ADI of spiromesifen and chromafenozide estimated in this study was 6.8333% and 0.5694% respectively in lettuce and 4.6000% and 0.2556% in perilla leaves. The previous study conducted by Chun and Kang^37^ claims that the risk of the target pesticide is considered relatively low when %ADI value is less than 10%. TMDI, the sum of %ADI of spiromesifen and chromafenozide, was 67.4925% and 3.4315%, correspondingly. The potential risk to consumers occurs when the TMDI ratio exceeds 80%. Thus, no significant effects of spiromesifen and chromafenozide residues were found in lettuce and perilla leaves on human health due to relatively low values^37^.

**Table 4:** TMDI and %ADI of spiromesifen and chromafenozide in lettuce and perilla leaves.

Insecticides	Crops	MRL (mg/kg)	Daily intake (g/day)	EDI (mg/kg)	%ADI	TMDI (%)
Spiromesifen	Lettuce	30 (recommended)	6.15	0.123	6.8333	67.4925
	Perilla leaves	30	2.76	0.0828	4.600	
Chromafenozide	Lettuce	15	6.15	0.0923	0.5694	3.4315
	Perilla leaves	15	2.76	0.0414	0.2556	

*MRL* maximum residue limit, *EDI* estimated daily intake, *TMDI* theoretical maximum daily intake.

## Conclusion

This study determined the dissipation and residual characteristics of spiromesifen and chromafenozid in lettuce and perilla leaves using LC-MS/MS. The result indicated that both insecticides dissipated rapidly in both matrices under greenhouse conditions. The spiromesifen and chromafenozide residues in lettuce and perilla leaves dissipated following the first-order kinetics model with the half-lives of 2.89 and 1.69 days in lettuce and 4.25 and 6.30 days in perilla leaves, respectively. The dissipation of both tested insecticides in lettuce was faster than that in perilla leaves. The safety assessment, based upon the TMDI, demonstrated that following the recommended guidelines for insecticides used in this study, lettuce and perilla leaves could be consumed safely without health problems. The data provided by this study can contribute to the management and regulation of pesticide use in lettuce and perilla as well as help in the sustainable production of minor crops in Republic of Korea.

## Materials and methods

### Chemicals and materials

Analytical standard (> 99% purity) of spiromesifen, BSN2060-enol, and chromafenozide were purchased from AB Solution Co., Ltd., Hwaseong-si, Gyeonggi-do, Republic of Korea. HPLC grade water and acetonitrile were supplied by Merck, Darmstadt, Germany. QuEChERS kit (4.0 g magnesium sulfate, 1.0 g sodium chloride, 1.0 g sodium citrate tribasic dihydrate, 0.5 g disodium citrate sesquihydrate) were obtained from Phenomenex, California, USA. Individual stock solutions of the target compounds were prepared in acetonitrile and stored at − 20 °C before use.

### Field experiments

The trials were carried out in a greenhouse farm during the season 2018 at two different sites (with approximately 24 km distance between both sites) located in Chuncheon and Hongcheon-gun, Gangwon-do, Republic of Korea following the method described by the Organization for Economic Co-operation and Development (OECD)^38^. The field test of lettuce (*Latuca sativa* L.) crop was conducted in Chuncheon city, and perilla (*Perilla frutescens* (L.) Britton) crop in Hongcheon city. The area of each field was divided into two plots (treatment and control). The treatment plots were further divided into three replicates (subplots 33 m^2^). The control plot was separated by a buffer zone of 3 m^2^ from the treated site. To minimize spray overlap, buffer zones (1 m) were set up between subplots. The commercial products of spiromesifen 20% SC diluted 2000 times and chromafenozide 5% EC diluted 1000 times were sprayed twice at 7-days intervals using an automatic sprayer. After the second spray samples (lettuce and perilla leaves) were collected from each subplot at 0 (2 h after spraying), 1, 3, 5, and 7 days according to the Korean RDA^23^ method. Thirty samples 1.0 kg each from the collected crop were placed in polyethylene bag and labeled. After collection, the samples were transported to the laboratory, where they were chopped and homogenized. The homogenized samples were kept frozen at − 20 °C until analysis.

We confirm all plant samples used in the current work comply with the IUCN Policy Statement on Research Involving Species at Risk of Extinction and the Convention on the Trade in Endangered Species of Wild Fauna and Flora.

### Samples pretreatment

A QuEChERS method was used for the extraction of the targeted compounds from lettuce and perilla leaves. A 10 g of previously homogenized samples were weighed into a 50 mL polypropylene centrifuge tube and mixed with 10 mL of water followed by 10 mL of acetonitrile. The samples were shaken at 1500 rpm in a shaker machine for 10 min. Then commercial QuEChERS kit was added, and the mixtures were shaken vigorously for 2 min in a shaker. Subsequently, the samples were centrifuged at 3584 g-force for 10 min. After centrifugation, the supernatant was filtered with a 0.22 μm membrane filter and transferred into the glass vial for instrumental analysis.

### LC-MS/MS analysis

Quantitative determination of the tested compounds was carried out by using HPLC system Dionex Ultimate 3000 (Thermo Science, USA) coupled with tandem mass spectrometry (MS/MS) (TSQ Quantum Access Max (Thermo Science, USA). Water (solvent A) and acetonitrile (solvent B) containing 0.1% formic acid and 5 mM ammonium format were used as mobile phase at a flow rate of 0.4 mL/min and injection volume 1.0 µL. To obtain desirable chromatographic peaks, two different instrumental conditions were used. The chromatographic separation of spiromesifen was separated by Capcell core-C18 (2.1 mm I.D. × 150 mm × 2.7 μm, Shiseido Co., Ltd., Tokyo, Japan) and BSN2060-enol was performed by C18 column (Poroshell 120 SB-Ag, 2.1 mm I.D. × 100 mm × 2.7-μm, Agilent Technologies, Santa Clara, California, USA) with a gradient elution as follows (mobile phase B%): 0.0 min, 5.0%; 2.0 min 5%; 2.5 min, 95%; 6.0 min, 95%; 6.5 min, 5.0%; 10 min, 5.0%. Likewise, chromafenozide was separated by C18 column (Imtakt Unison UK-C18, 2.0 mm I.D. × 100 mm × 3.0-μm, Imtakt, Portland, USA) with a gradient elution as follows (mobile phase B%): 0.0 min, 5%; 1.0 min, 5.0%; 1.5 min, 90%; 5.0 min, 90%; 7.0 min, 5.0%; 10 min, 5.0%. An MS/MS system (TSQ quantum ultra, Thermo Science, USA) equipped with an electrospray ionization source operating in positive mode (ESI+) was used. The MS/MS parameters and selected product ions are shown in supplementary Tables S2 and S3.

### The calculation of spiromesifen total residues

The total residues in lettuce and perilla samples were calculated using Eq. (1)^23^.

Total residues of spiromesifen (mg/kg) = spiromesifen + (BSN2060 residue × 1.36). The conversion factor was calculated as follow;

1 $${1}.{36}\,{\text{(conversion}}\,{\text{factor)}} = \frac{{370.49\left( {{\text{spiromesifen}}\,{\text{MW}}} \right)}}{{272.34{ }\left( {{\text{BSN}}2060\,{\text{MW}}} \right)}}$$

where *MW* molecular weight.

### Initial deposition calculation

The initial residues of spiromesifen and chromafenozide deposition in lettuce and perilla leaves were calculated from 0-day according to Eq. (2) described by Kang et al.^12^ as follow;

2 $${\text{A }}\,({\text{mg}}/{\text{kg}}) = {\text{B(mg}}/{\text{kg)}} \times \frac{100}{{{\text{C}}({\text{\% }})}} \times \frac{1}{{\text{E}}} \times 1000$$

A: Initial residue (mg/kg), B: Residues (mg/kg) on 0 day, C: active ingredients, E: dilution factor.

### Method validation

The analytical method was validated in terms of different performance criteria such as linearity, accuracy, precision, and method limit of quantitation (MLOQ). Matrix-matched standards were used to construct the calibration curve by evaporating (0.01, 0.05, 0.1, 0.2, 0.5, 0.7 and 1.0 mg/kg) working solution (1 mL) and re-dissolved in the extract of control sample. The linearity of the matrix-matched calibration curve was evaluated by the values of the correlation coefficient (R^2^). The accuracy and precision were obtained in terms of recovery (70–120%) and repeatability (n = 3). The recoveries were determined by spiking the analytes at two concentrations levels (0.1 mg/L) and (0.5 mg/L) in 10 g control samples and were quantified by comparing the response of analytes in samples with response in calibration standard solutions prepared in matrix. The repeatability expressed as the relative standard deviation (RSD) of the analyzed samples was calculated from three repetitions. The MLOQ was calculated by Eq. (3) taking into consideration the following factors: the instrument limit of detection, volume of extraction solvent, injection volume, dilution factor, and sample amount^39,40^.

3 $${\text{MLOQ}}\,{\text{(mg}}/{\text{kg)}} = {\text{A(ng)}} \times \frac{{\text{B(mL)}}}{{{\text{C(}}\upmu {\text{L)}}}} \times \frac{{\text{D}}}{{\text{E(g)}}}$$

where A: instrument limit of detection, B: volume of extraction solvent, C: injection volume, D: dilution factor, E: sample amount.

### Half-life calculation

The dissipation patterns of spiromesifen and chromafenozide in lettuce and perilla leaves over time were found following the first-order kinetics model^28^. The half-life was determined by the following equation:

$${\text{C}}_{{\text{t}}} = {\text{C}}_{0} \times {\text{e}}^{{ - {\text{kt}}}} ,{\text{DT}}_{{{5}0}} = {\text{ln2}}/{\text{k}}$$

where C_t_ is the concentration of the insecticide, C_0_ represents the initial residue concentration of insecticide, t is the time (days) after insecticide application, and k is the constant rate.

### Safety assessment

In this study, the safety assessments (percent acceptable daily intake; %ADI) of the target insecticides that are consumed with lettuce and perilla leaves were calculated by the ratio of estimated daily intake (EDI) to acceptable daily intake (ADI). The EDI was calculated using insecticide concentration and average consumption of food commodities per person per day. In addition, the theoretical maximum daily intakes (TMDIs) of both insecticides were calculated using the maximum residue limits (MRLs) and average body weight (60 kg) of adults in Republic of Korea. TMDIs were calculated following the equation described by Kim et al.^41^.

$$\begin{aligned} & {\text{ADI (mg}}/{\text{person}}/{\text{day)}} = {\text{ADI}}\,({\text{mg}}/{\text{kg}}/{\text{body weight}}/{\text{day}})\,{\text{of target insecticide}} \times {\text{6}}0\,({\text{average body weight}}) \\ & {\text{EDI (mg}}/{\text{kg}}/{\text{person)}} = {\text{concentration of target insecticide (mg}}/{\text{kg)}} \times {\text{ daily food intake (g)}} \\ & \% {\text{ADI}} = {\text{EDI}}/{\text{ADI}} \times {\text{1}}00 \\ & {\text{TMDI}}\% = \sum \% {\text{ADI of all registered crops}} \\ \end{aligned}$$

## Supplementary Information

Supplementary Information.

## Data Availability

Data are presented in the manuscript and available as support information.

## Abbreviations

ADI: Acceptable daily intake
AI: Active ingredients
EC: Emulsifiable concentrate
EDI: Estimated daily intake
ESI: Electrospray ionization
KHIDI: Korean Health Industry Development Institute
LC-MS/MS: Liquid chromatography tandem mass spectrometry
MFDS: Ministry of Food and Drug Safety
MLOQ: Method limits of quantification
MRL: Maximum residue limit
OECD: Organization for Economic Co-operation and Development
PHI: Pre-harvest interval
QuEChERS: Quick, easy, cheap, effective, rugged, and safe
RDA: Rural Development Administration
RSD: Relative standard deviation
SC: Suspension concentrate
TMDI: Theoretical maximum daily intake
WP: Wettable powder
