# Supplementary material for: Residual characteristics and safety assessment of the insecticides spiromesifen and chromafenozide in lettuce and perilla
Source: Sci Rep. 2022 Mar 18;12:4675. doi: 10.1038/s41598-022-08532-2 (PMC8933456; doi:10.1038/s41598-022-08532-2)
Supplement: Supplementary file 1 — Supplementary Information. [file 41598_2022_8532_MOESM1_ESM.docx]

**Supplementary information;**

**Residual characteristics and safety assessment of the insecticides spiromesifen and chromafenozide in lettuce and perilla**

**Syed Wasim Sardar1^+^, Geon-doo Byeon^1+^, Jeong-Yoon Choi^1^, Hun-Ju Ham2, Abd Elaziz Sulieman Ahmed Ishag^1&^3, Jang-Hyun Hur^*1^**

Department of Biological Environment, Kangwon National University, Chuncheon 24341, Republic of Korea

2Environmentally Friendly Agricultural Products Safety Center, Chuncheon 24341, Republic of Korea

3Department of Crop Protection, University of Khartoum, Khartoum North, Shambat 13314, Sudan

Corresponding author*: E-mail: [hurpub303@gmail.com](file:///H:\Kundo_Wasim_paper\Paper_draft%20new\hurpub303@gmail.com); Tel: +82-(0)33-257-6441, Fax: +82-(0)33-259-5563

+These authors contributed equally to this work

**Table S1.** Recoveries of insecticides in lettuce and perilla leaves

| Insecticides | Crops | Fortification level  (mg/kg) | Recoveries (%) | | |
| --- | --- | --- | --- | --- | --- |
|  |  |  | ^*^Rep 1 | Rep 2 | Rep 3 |
| Spiromesifen | Lettuce | 0.1  0.5 | 100.2  88.2 | 95.8  101.9 | 101.6  93.5 |
|  | Perilla leaves | 0.1  0.5 | 92.7  79.2 | 99.9  78.0 | 91.1  86.6 |
| BSN2060-enol | Lettuce | 0.1  0.5 | 98.9  103.2 | 97.3  99.7 | 99.9  97.4 |
|  | Perilla leaves | 0.1  0.5 | 77.8  80.2 | 83.2  79.9 | 80.7  87.5 |
| Chromafenozide | Lettuce | 0.1  0.5 | 100.4  98.5 | 99.7  95.6 | 99.7  100.8 |
|  | Perilla leaves | 0.1  0.5 | 101.7  104.7 | 104.9  113.5 | 99.4  105.4 |

^*^Repetition

**Table S2.** LC-MS/MS operating conditions for the analysis of spiromesifen and its metablite BSN2060-enol in lettuce and perilla leaves

| **HPLC** | | **Dionex Ultimate 3000 (Thermo Science, USA)** | | | | | | | |
| --- | --- | --- | --- | --- | --- | --- | --- | --- | --- |
| Column | | Poroshell 120 SB-Ag (2.1 mm I.D.×100 mm×2.7 μm) | | | | | | | |
| Column Temp. | | 45℃ | | | | | | | |
| Gradient condition | | Flow(mL/min.) | | Time | A (%) | | B (%) | | |
|  |  | 0.4 | | 0.0 | 95 | | 5 | | |
|  |  |  |  | 2.0 | 95 | | 5 | | |
|  |  |  |  | 2.5 | 5 | | 95 | | |
|  |  |  |  | 6.0 | 5 | | 95 | | |
|  |  |  |  | 6.5 | 95 | | 5 | | |
|  |  |  |  | 10.0 | 95 | | 5 | | |
|  | |  | |  |  | |  | | |
| **MS/MS** | | **TSQ Quantum Access Max (Thermo Science, USA)** | | | | | | | |
| Ionization mode | | ESI+ | | | | | | | |
| Spray voltage | | 4,000 V | | | | | | | |
| Capillary Temp. | | 320℃ | | | | | | | |
| Vaporizer Temp. | | 350℃ | | | | | | | |
| Sheath Gas  Pressure (N2) | | 35 units | | | | | | | |
| Aux Gas  Pressure (N2) | | 10 units | | | | | | | |
| Collision gas & pressure | | Argon, 1.5 Torr | | | | | | | |
| Scan event | | SRM (Selected Reaction Monitoring) mode | | | | | | | |
| Pesticides | | Precursor ion | Product ion | | CE | | Retention time (min.) |  |  |
| Spiromesifen | | 371.200 | 255.150 | | 23 | | 6.03 |  |  |
|  |  |  | 273.160 | | 11 | |  |  |  |
| BSN2060-enol | | 273.160 | 131.190 | | 29 | | 5.53 |  |  |
|  |  |  | 187.140 | | 18 | |  |  |  |

**Table S3.** LC-MS/MS operating conditions for the analysis of chromafenozide in lettuce and perilla leaves

| **HPLC** | | **Dionex Ultimate 3000 (Thermo Science, USA)** | | | | | | |
| --- | --- | --- | --- | --- | --- | --- | --- | --- |
| Column | | Imtakt Unison UK-C_18_ (2.0 mm I.D.×100 mm×3.0 μm) | | | | | | |
| Column Temp. | | 45℃ | | | | | | |
| Gradient condition | | Flow(mL/min.) | | Time | A (%) | | B (%) | |
|  |  | 0.4 | | 0.0 | 95 | | 5 | |
|  |  |  |  | 1.0 | 95 | | 5 | |
|  |  |  |  | 1.5 | 10 | | 90 | |
|  |  |  |  | 5.0 | 10 | | 90 | |
|  |  |  |  | 7.0 | 95 | | 5 | |
|  |  |  |  | 10.0 | 95 | | 5 | |
|  | |  | |  |  | |  | |
| **MS/MS** | | **TSQ Quantum Access Max (Thermo Science, USA)** | | | | | | |
| Ionization mode | | ESI+ | | | | | | |
| Spray voltage | | 3,500 V | | | | | | |
| Capillary Temp. | | 270℃ | | | | | | |
| Vaporizer Temp. | | 320℃ | | | | | | |
| Sheath Gas  Pressure (N2) | | 35 units | | | | | | |
| Aux Gas  Pressure (N2) | | 10 units | | | | | | |
| Collision gas & pressure | | Argon, 1.5 Torr | | | | | | |
| Scan event | | SRM (Selected Reaction Monitoring) mode | | | | | | |
| Pesticide | | Precursor ion | Product ion | | CE | | Retention time (min.) |  |
| Chromafenozide | | 395.194 | 147.134 | | 43 | | 6.21 |  |
|  |  |  | 175.120 | | 19 | |  |  |

| Spiromesifen | BSN2060-enol | | Chromafenozide | |
| --- | --- | --- | --- | --- |
| 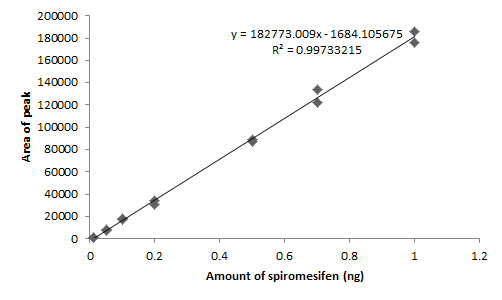 | 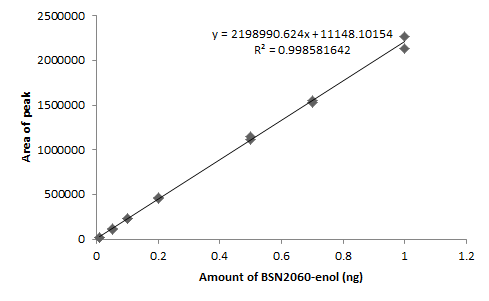 | | 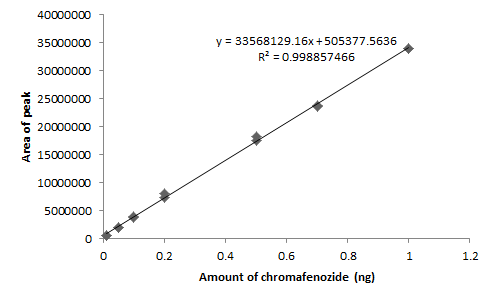 | |
| \| Fig. S1. Matrix matched calibration curve of target analytes in lettuce (*Lactuca sativa* L.) \| \| --- \| | | | | |
| Spiromesifen | | BSN2060-enol | | Chromafenozide |
| 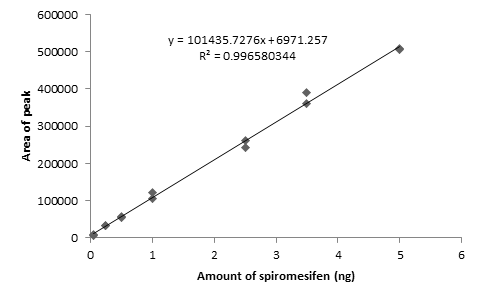 | | 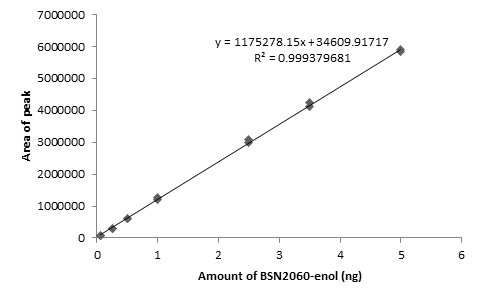 | | 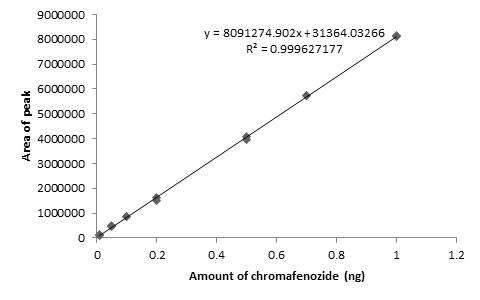 |
| \| Fig. Fig S2. Matrix matched calibration curve of target analytes in perilla leaves (*Perilla frutescens* var. *japonica* Hara) \| \| --- \| | | | | |
